# The genome sequence of a bird blowfly, *Protocalliphora azurea* (Fallén,1817)

**DOI:** 10.12688/wellcomeopenres.18872.1

**Published:** 2023-02-09

**Authors:** Steven Falk, Darwin Tree of Life Barcoding collective, Wellcome Sanger Institute Tree of Life programme, Wellcome Sanger Institute Scientific Operations: DNA Pipelines collective, Tree of Life Core Informatics collective, Olga Sivell, Darwin Tree of Life Consortium

**Affiliations:** 1 Independent researcher, Kenilworth, Warwickshire, UK; 2 Natural History Museum, London, UK

**Keywords:** Protocalliphora azurea, bird blowfly, genome sequence, chromosomal, Diptera

## Abstract

We present a genome assembly from an individual male *Protocalliphora azurea* (a bird blowfly; Arthropoda; Insecta; Diptera; Calliphoridae). The genome sequence is 874 megabases in span. Most of the assembly is scaffolded into seven chromosomal pseudomolecules, including the assembled X and Y sex chromosomes. The mitochondrial genome has also been assembled and is 15.7 kilobases in length. Gene annotation of this assembly on Ensembl identified 11,936 protein coding genes.

## Species taxonomy

Eukaryota; Metazoa; Ecdysozoa; Arthropoda; Hexapoda; Insecta; Pterygota; Neoptera; Endopterygota; Diptera; Brachycera; Muscomorpha; Oestroidea; Calliphoridae; Chrysomyinae; *Protocalliphora*; *Protocalliphora azurea* (Fallén, 1817) (NCBI:txid226133).

## Background

*Protocalliphora azurea* (Diptera, Calliphoridae) is easily recognisable in the field as a shiny metallic fly with dusting and longitudinal stripes on the thorax. The males are dark blue, while females are bottle-green ( Rognes, 1991; Sivell, 2021). This blowfly is common and widely distributed in England and Wales, but uncommon and scattered in Scotland. It has been recorded from March to October ( Highland Biological Recording Group, 2021; Sivell, 2021). This species is attracted to flowers, honeydew, rotting fruit, carrion, manure and bird droppings.

*Protocalliphora azurea* is commonly referred to as a bird blowfly due to its biology. While adult flies feed mainly on flowers, the larvae are obligatory external parasites of birds. They infest variety of bird species ( Owen, 1954; Owen & Ash, 1955; Zumpt, 1965). The larvae suck blood of nestlings causing primary myiasis. Usually only a few larvae are involved and the host recovers, although death from blood loss may occur. Subsequently, the birds may also become infested with other fly species, *e.g*. *Protophormia terraenovae* (species from the same subfamily as *P. azurea*: Chrysomyinae) in secondary myiasis ( van Emden, 1954; Zumpt, 1965).

*Protocalliphora azurea* is oviparous. Larvae feed approximately for one week, then pupate in the host’s nest. Adult flies emerge after 7–12 days ( Draber-Mońko, 2004). Duration of development is dependent on temperature and other conditions ( Draber-Mońko, 2004; Zumpt, 1965). The pupae are often infested by the parasitoid wasp *Nasonia vitripennis* (Walker, 1836) ( Draber-Mońko, 1997). The fly overwinters as adult ( Draber-Mońko, 2004). It has been reared from bird nests, also found in houses and in insect hotels ( Sivell, 2021; van Emden, 1954).

The high-quality genome assembly described here, generated as part of the Darwin Tree of Life Project, is the first reported for *Protocalliphora azurea*. It will aid research on the taxonomy, biology and ecology of the species.

## Genome sequence report

**Figure 1.**
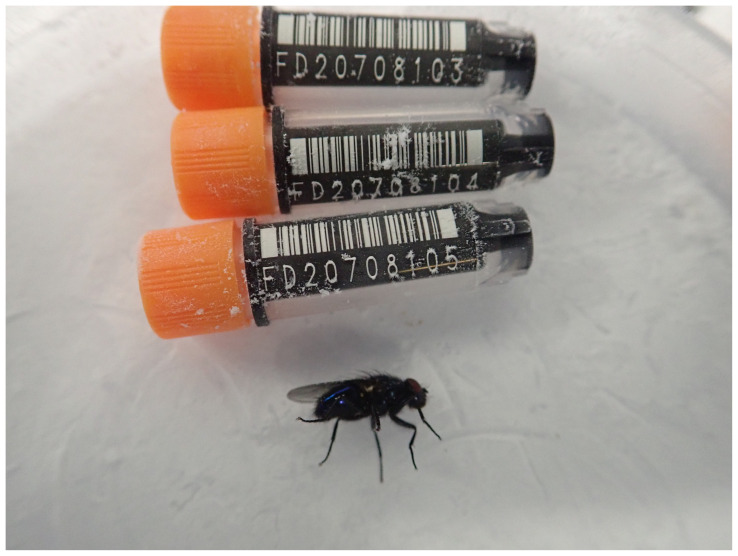
Image of the *Protocalliphora azurea* (idProAzur1) specimen used for genome sequencing.

The genome was sequenced from one male *Protocalliphora azurea* specimen ( Figure 1) collected from Wytham Woods, Oxfordshire (biological vice-county: Berkshire), UK (latitude 51.769, longitude –1.34). A total of 39-fold coverage in Pacific Biosciences single-molecule HiFi long reads and 78-fold coverage in 10X Genomics read clouds were generated. Primary assembly contigs were scaffolded with chromosome conformation Hi-C data. Manual assembly curation corrected 190 missing joins or mis-joins and removed one haplotypic duplications, reducing the scaffold number by 81.3% and increasing the scaffold N50 by 7.15%.

**Figure 2.**
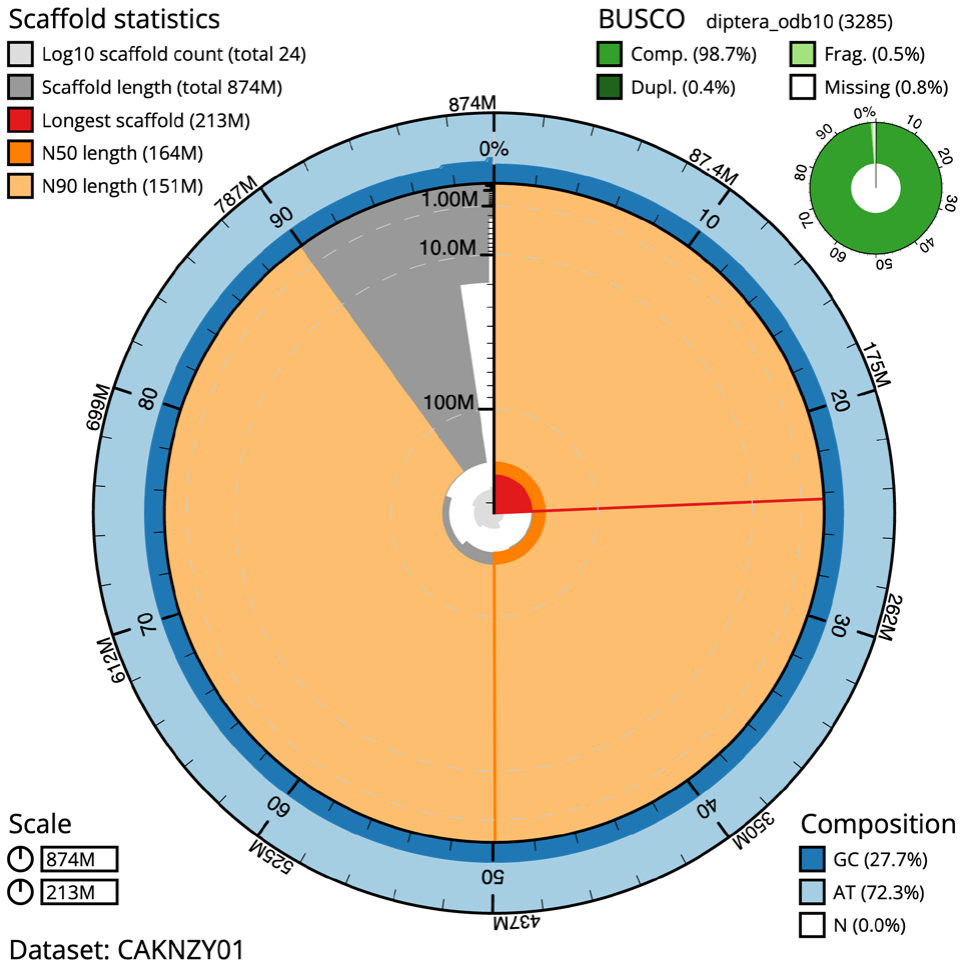
Genome assembly of *Protocalliphora azurea*, idProAzur1.1: metrics. The BlobToolKit Snailplot shows N50 metrics and BUSCO gene completeness. The main plot is divided into 1,000 size-ordered bins around the circumference with each bin representing 0.1% of the 874,252,225 bp assembly. The distribution of scaffold lengths is shown in dark grey with the plot radius scaled to the longest scaffold present in the assembly (212,808,213 bp, shown in red). Orange and pale-orange arcs show the N50 and N90 scaffold lengths (164,199,054 and 151,389,329 bp), respectively. The pale grey spiral shows the cumulative scaffold count on a log scale with white scale lines showing successive orders of magnitude. The blue and pale-blue area around the outside of the plot shows the distribution of GC, AT and N percentages in the same bins as the inner plot. A summary of complete, fragmented, duplicated and missing BUSCO genes in the diptera_odb10 set is shown in the top right. An interactive version of this figure is available at https://blobtoolkit.genomehubs.org/view/idProAzur1.1/dataset/CAKNZY01/snail.

The final assembly has a total length of 874.2 Mb in 23 sequence scaffolds with a scaffold N50 of 164.2 Mb ( Table 1). Most (99.96%) of the assembly sequence was assigned to seven chromosomal-level scaffolds, representing five autosomes and the X and Y sex chromosomes. Chromosome-scale scaffolds confirmed by the Hi-C data are named in order of size ( Figure 2– Figure 5; Table 2). The assembly has a BUSCO 5.3.2 ( Manni *et al.*, 2021) completeness of 98.7% (single 98.4%, duplicated 0.4%) using the OrthoDB v10 Diptera reference set ( *n*=3,285). While not fully phased, the assembly deposited is of one haplotype. Contigs corresponding to the second haplotype have also been deposited.

**Table 1.** Genome data for *Protocalliphora azurea*, idProAzur1.1.

Project accession data		
Assembly identifier	idProAzur1.1	
Species	*Protocalliphora azurea*	
Specimen	idProAzur1	
NCBI taxonomy ID	226133	
BioProject	PRJEB48588	
BioSample ID	SAMEA7746778	
Isolate information	male, whole organism	
Assembly metrics		*Benchmark*
BUSCO *	C:98.7%[S:98.4%,D:0.4%], F:0.5%,M:0.8%,n:3,285	*C ≥ 95%*
Percentage of assembly mapped to chromosomes	99.96%	*≥ 95%*
Sex chromosomes	X and Y chromosomes	*localised homologous pairs*
Organelles	Mitochondrial genome assembled	*complete single alleles*
Raw data accessions		
PacificBiosciences SEQUEL II	ERR7254656, ERR7254657	
10X Genomics Illumina	ERR7253241–ERR7253244	
Hi-C Illumina	ERR7253240	
PolyA RNA-Seq Illumina	ERR10123665	
Genome assembly		
Assembly accession	GCA_932274085.1	
*Accession of alternate haplotype*	GCA_932273785.1	
Span (Mb)	874.2	
Number of contigs	411	
Contig N50 length (Mb)	8.4	
Number of scaffolds	23	
Scaffold N50 length (Mb)	164.2	
Longest scaffold (Mb)	212.8	
**Genome annotation**		
Number of protein-coding genes	11,936	
Non-coding genes	1,744	
Gene transcripts	18,968	

* BUSCO scores based on the diptera_odb10 BUSCO set using v5.3.2. C = complete [S = single copy, D = duplicated], F = fragmented, M = missing, n = number of orthologues in comparison. A full set of BUSCO scores is available at https://blobtoolkit.genomehubs.org/view/idProAzur1.1/dataset/CAKNZY01/busco.

**Figure 3.**
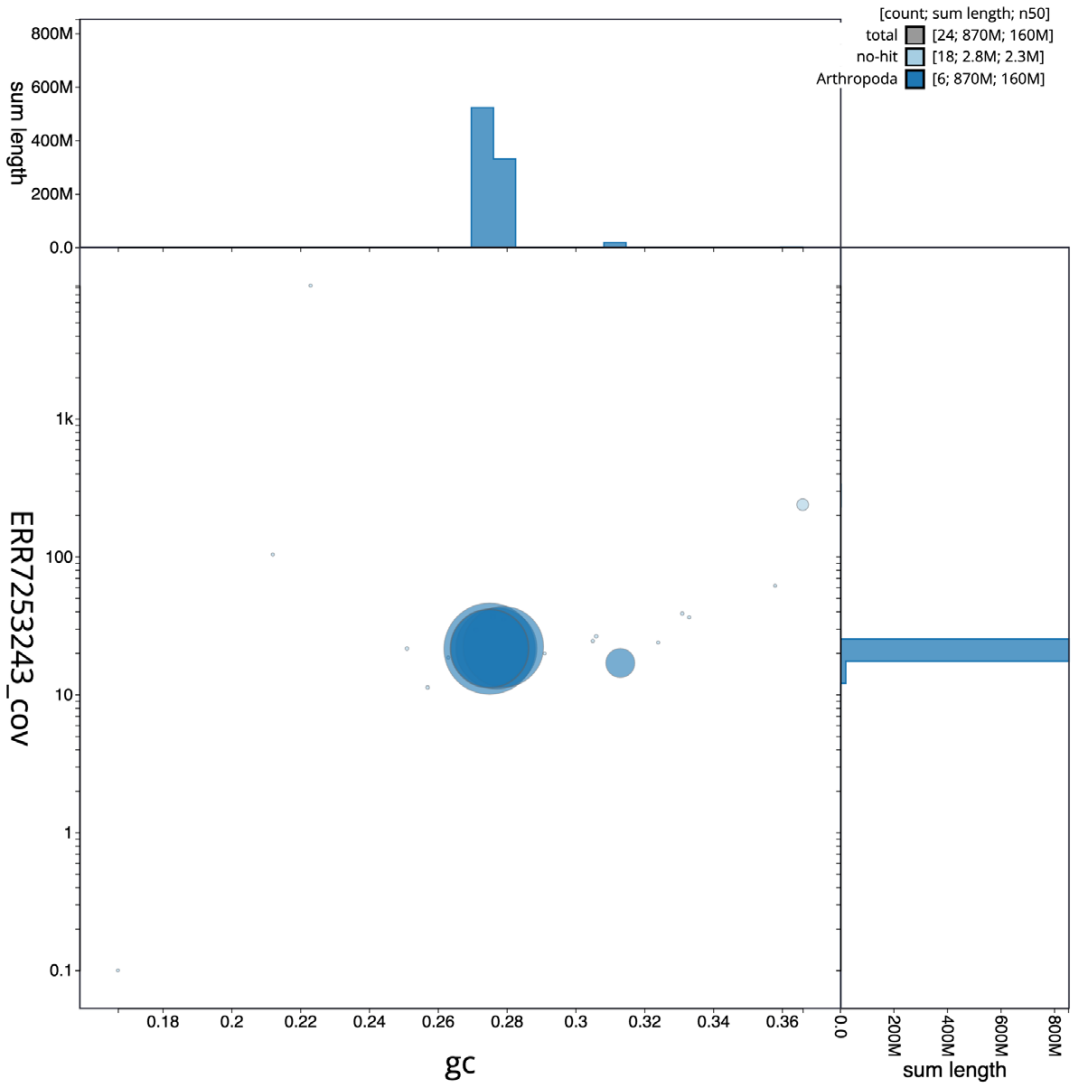
Genome assembly of *Protocalliphora azurea*, idProAzur1.1: GC coverage. BlobToolKit GC-coverage plot. Scaffolds are coloured by phylum. Circles are sized in proportion to scaffold length. Histograms show the distribution of scaffold length sum along each axis. An interactive version of this figure is available at https://blobtoolkit.genomehubs.org/view/idProAzur1.1/dataset/CAKNZY01/blob.

**Figure 4.**
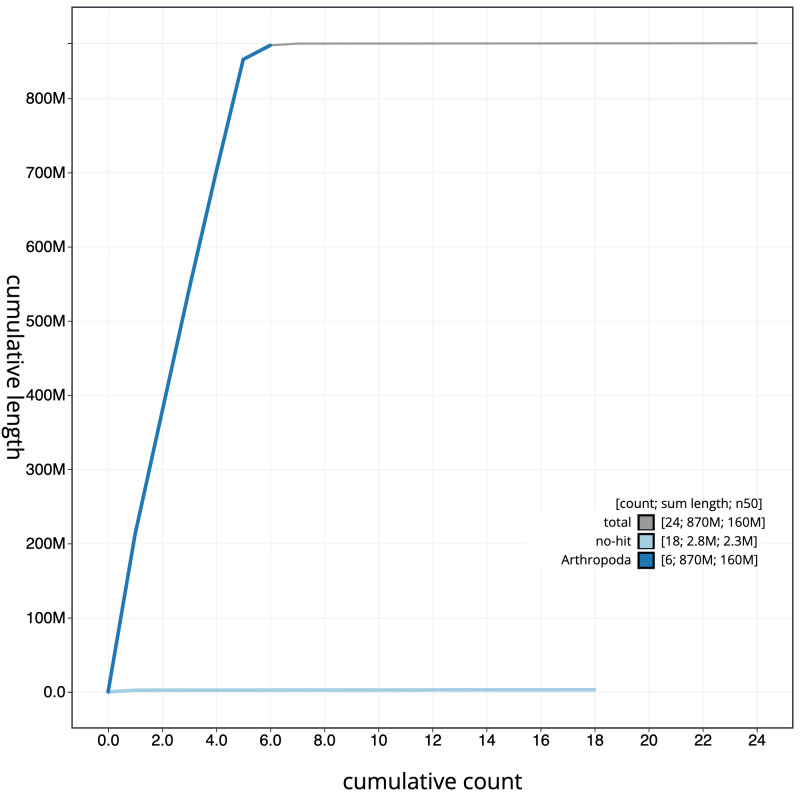
Genome assembly of *Protocalliphora azurea*, idProAzur1.1: cumulative sequence. BlobToolKit cumulative sequence plot. The grey line shows cumulative length for all scaffolds. Coloured lines show cumulative lengths of scaffolds assigned to each phylum using the buscogenes taxrule. An interactive version of this figure is available at https://blobtoolkit.genomehubs.org/view/idProAzur1.1/dataset/CAKNZY01/cumulative.

**Figure 5.**
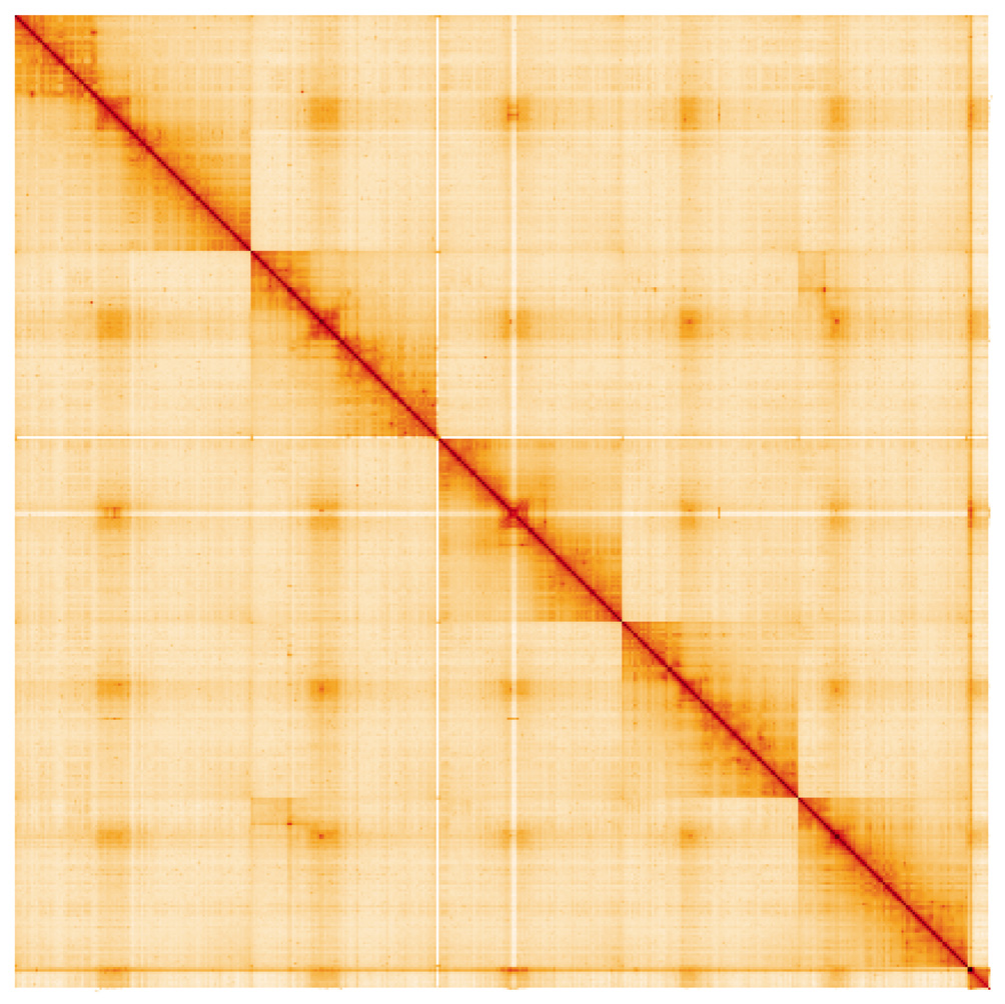
Genome assembly of *Protocalliphora azurea*, idProAzur1.1: Hi-C contact map. Hi-C contact map of the idProAzur1.1 assembly, visualised using HiGlass. Chromosomes are shown in order of size from left to right and top to bottom. An interactive version of this figure may be viewed at https://genome-note-higlass.tol.sanger.ac.uk/l/?d=ftusVv38SaqwPwj6_Ij3OA.

**Table 2.** Chromosomal pseudomolecules in the genome assembly of *Protocalliphora azurea*, idProAzur1.

INSDC accession	Chromosome	Size (Mb)	GC%
OW026519.1	1	212.81	27.5
OW026520.1	2	166.18	27.7
OW026521.1	3	164.2	27.9
OW026522.1	4	157.91	27.5
OW026523.1	5	151.39	27.5
OW026524.1	X	18.98	31.3
OW026525.1	Y	2.32	36.6
OW026526.1	MT	0.02	22.3
-	unplaced	0.45	28.4

### Genome annotation report

The idProAzur1.1 genome assembly was annotated using the Ensembl rapid annotation pipeline ( Table 1; https://rapid.ensembl.org/Protocalliphora_azurea_GCA_932274085.1/). The resulting annotation includes 18,968 transcribed mRNAs from 11,936 protein-coding and 1,744 non-coding genes.

## Methods

### Sample acquisition and nucleic acid extraction

A male *Protocalliphora azurea* (idProAzur1) was caught using a net in Wytham Woods, Oxfordshire (biological vice-county: Berkshire), UK (latitude 51.769, longitude –1.34) on 20 August 2020. The specimen was collected and identified by Steven Falk (independent researcher), and was then snap-frozen on dry ice.

DNA was extracted at the Tree of Life laboratory, Wellcome Sanger Institute (WSI). The idProAzur1 sample was weighed and dissected on dry ice with head tissue set aside for Hi-C sequencing. Thorax was disrupted using a Nippi Powermasher fitted with a BioMasher pestle. High molecular weight (HMW) DNA was extracted using the Qiagen MagAttract HMW DNA extraction kit. Low molecular weight DNA was removed from a 20 ng aliquot of extracted DNA using 0.8X AMpure XP purification kit prior to 10X Chromium sequencing; a minimum of 50 ng DNA was submitted for 10X sequencing. HMW DNA was sheared into an average fragment size of 12–20 kb in a Megaruptor 3 system with speed setting 30. Sheared DNA was purified by solid-phase reversible immobilisation using AMPure PB beads with a 1.8X ratio of beads to sample to remove the shorter fragments and concentrate the DNA sample. The concentration of the sheared and purified DNA was assessed using a Nanodrop spectrophotometer and Qubit Fluorometer and Qubit dsDNA High Sensitivity Assay kit. Fragment size distribution was evaluated by running the sample on the FemtoPulse system.

RNA was extracted from abdomen tissue of idProAzur1 in the Tree of Life Laboratory at the WSI using TRIzol, according to the manufacturer’s instructions. RNA was then eluted in 50 μl RNAse-free water and its concentration assessed using a Nanodrop spectrophotometer and Qubit Fluorometer using the Qubit RNA Broad-Range (BR) Assay kit. Analysis of the integrity of the RNA was done using Agilent RNA 6000 Pico Kit and Eukaryotic Total RNA assay.

### Sequencing

Pacific Biosciences HiFi circular consensus and 10X Genomics read cloud DNA sequencing libraries were constructed according to the manufacturers’ instructions. Poly(A) RNA-Seq libraries were constructed using the NEB Ultra II RNA Library Prep kit. DNA and RNA sequencing were performed by the Scientific Operations core at the WSI on Pacific Biosciences SEQUEL II (HiFi) and Illumina NovaSeq 6000 (10X and RNA-Seq) instruments. Hi-C data were also generated from head tissue of idProAzur1 using the Arima v2 kit and sequenced on the Illumina NovaSeq 6000 instrument.

#### Genome assembly

Assembly was carried out with Hifiasm ( Cheng *et al.*, 2021) and haplotypic duplication was identified and removed with purge_dups ( Guan *et al.*, 2020). One round of polishing was performed by aligning 10X Genomics read data to the assembly with Long Ranger ALIGN, calling variants with freebayes ( Garrison & Marth, 2012). The assembly was then scaffolded with Hi-C data ( Rao *et al.*, 2014) using YaHS ( Zhou *et al.,* 2022). The assembly was checked for contamination as described previously ( Howe *et al.*, 2021). Manual curation was performed using HiGlass ( Kerpedjiev *et al.*, 2018) and Pretext ( Harry, 2022). The mitochondrial genome was assembled using MitoHiFi ( Uliano-Silva *et al.*, 2022), which performed annotation using MitoFinder ( Allio *et al.*, 2020). The genome was analysed and BUSCO scores generated within the BlobToolKit environment ( Challis *et al.*, 2020). Table 3 contains a list of all software tool versions used, where appropriate.

**Table 3.** Software tools and versions used.

Software tool	Version	Source
BlobToolKit	3.2.6	Challis *et al.*, 2020
freebayes	1.3.1-17-gaa2ace8	Garrison & Marth, 2012
Hifiasm	0.15.3	Cheng *et al.*, 2021
HiGlass	1.11.6	Kerpedjiev *et al.*, 2018
Long Ranger ALIGN	2.2.2	https://support.10xgenomics.com/genome-exome/software/pipelines/latest/advanced/other-pipelines
MitoHiFi	2	Uliano-Silva *et al.*, 2022
PretextView	0.2	Harry, 2022
purge_dups	1.2.3	Guan *et al.*, 2020
YaHS	1	Zhou *et al*., 2022

#### Genome annotation

The Ensembl gene annotation system ( Aken *et al.*, 2016) was used to generate annotation for the *Protocalliphora azurea* assembly GCA_932274085.1. Annotation was created primarily through alignment of transcriptomic data to the genome, with gap filling via protein to-genome alignments of a select set of proteins from UniProt ( UniProt Consortium, 2019).

#### Ethics/compliance issues

The materials that have contributed to this genome note have been supplied by a Darwin Tree of Life Partner. The submission of materials by a Darwin Tree of Life Partner is subject to the Darwin Tree of Life Project Sampling Code of Practice. By agreeing with and signing up to the Sampling Code of Practice, the Darwin Tree of Life Partner agrees they will meet the legal and ethical requirements and standards set out within this document in respect of all samples acquired for, and supplied to, the Darwin Tree of Life Project. Each transfer of samples is further undertaken according to a Research Collaboration Agreement or Material Transfer Agreement entered into by the Darwin Tree of Life Partner, Genome Research Limited (operating as the Wellcome Sanger Institute), and in some circumstances other Darwin Tree of Life collaborators.

## Data Availability

European Nucleotide Archive: *Protocalliphora azurea (*bird blowfly). Accession number PRJEB48588; https://identifiers.org/ena.embl/PRJEB48588. ( Wellcome Sanger Institute, 2022). The genome sequence is released openly for reuse. The *Protocalliphora azurea* genome sequencing initiative is part of the Darwin Tree of Life (DToL) project. All raw sequence data and the assembly have been deposited in INSDC databases. Raw data and assembly accession identifiers are reported in Table 1.

## Author information

Members of the University of Oxford and Wytham Woods Genome Acquisition Lab are listed here: https://doi.org/10.5281/zenodo.4789928.

Members of the Darwin Tree of Life Barcoding collective are listed here: https://doi.org/10.5281/zenodo.4893703.

Members of the Wellcome Sanger Institute Tree of Life programme are listed here: https://doi.org/10.5281/zenodo.4783585.

Members of Wellcome Sanger Institute Scientific Operations: DNA Pipelines collective are listed here: https://doi.org/10.5281/zenodo.4790455.

Members of the Tree of Life Core Informatics collective are listed here: https://doi.org/10.5281/zenodo.5013541.

Members of the Darwin Tree of Life Consortium are listed here: https://doi.org/10.5281/zenodo.4783558.
