# Supplementary material for: The genome sequence of a bird blowfly, Protocalliphora azurea (Fallén,1817)
Source: Wellcome Open Res. 2023 Feb 9;8:67. [Version 1] doi: 10.12688/wellcomeopenres.18872.1 (PMC10521116; doi:10.12688/wellcomeopenres.18872.1)
Supplement: Peer review history

# Reviewer report: Reviewer response for version 1

**DOI:** 10.21956/wellcomeopenres.20926.r65700

**Published:** 2023-09-13

**Authors:** Sanjay Kumar Pradhan

**Affiliations:** 1 University of Agricultural Sciences, Bangalore, India

The manuscript is well structured, and all the methodologies are clearly mentioned.

1. Why was only the male fly sampled? Authors should explain why a single fly was sampled, rather than having some replications.
2. What taxonomic and ecological studies details are available currently in the domain, what are the loopholes the author finds regarding available information about *Protocalliphora azurea, *and how will the current findings fill the gap?”

Are sufficient details of methods and materials provided to allow replication by others?

Yes

Is the rationale for creating the dataset(s) clearly described?

Yes

Are the datasets clearly presented in a useable and accessible format?

Yes

Are the protocols appropriate and is the work technically sound?

Yes

Reviewer Expertise:

Gene Editing, Insect metagenomics

I confirm that I have read this submission and believe that I have an appropriate level of expertise to confirm that it is of an acceptable scientific standard.

# Reviewer report: Reviewer response for version 1

**DOI:** 10.21956/wellcomeopenres.20926.r65687

**Published:** 2023-09-11

**Authors:** Andrew D Sweet

**Affiliations:** 1 Arkansas State University, Jonesboro, Arkansas, USA

The authors sequenced, assembled, and annotated the genome and mitogenome from an individual male specimen of a species of bird blowfly (Protocalliphora azurae) using a combination of PacBio HiFi, 10X, RNA-Seq, and Hi-C data. 

 This genome report clearly lays out the approaches and assembly statistics for a species of bird blowfly. The chromosome-level assembly seems of high quality. I have a few comments that I think can help improve and clarify the report.

1. Can you provide more details about the manual assembly curation? How exactly did you correct for missing joins and haplotypic duplications?
2. Is it possible to use a clearer image of the organism for Figure 1? I understand the use of having images of the vials, but it is difficult to see features of the adult fly.
3. It is unclear you utilized RNA-Seq until the Methods section. I recommend including this information at the beginning of the Genome sequence report section.
4. The mitochondrial genome did not have annotations associated with the GenBank accession provided, so I was unable to assess the mitogenome annotation. Please ensure the annotation details are available with the GenBank record.
5. GC-content seems to be on the low side, although not the lowest for insects. Was the nucleotide content unsurprising for this taxon? 

Minor comments:

 Background:

 Paragraph 2, Line 2, “They infest a variety…”

 Paragraph 3, Line 4: “overwinters as an adult”

Are sufficient details of methods and materials provided to allow replication by others?

Partly

Is the rationale for creating the dataset(s) clearly described?

Yes

Are the datasets clearly presented in a useable and accessible format?

Partly

Are the protocols appropriate and is the work technically sound?

Partly

Reviewer Expertise:

Evolutionary genomics, mitogenomics, host-parasite coevolution, phylogenomics, population genomics, ornithology, entomology

I confirm that I have read this submission and believe that I have an appropriate level of expertise to confirm that it is of an acceptable scientific standard.

# Reviewer report: Reviewer response for version 1

**DOI:** 10.21956/wellcomeopenres.20926.r60239

**Published:** 2023-06-15

**Authors:** Xuan Li

**Affiliations:** 1 Uppsala University, Uppsala, Sweden

In the article, the authors present a chromosomal-level assembly of male genome of the blowfly, *Protocalliphora azurea. *Collected data (i.e. Pacbio Hifi, 10X reads and Hi-C reads) are sufficient and the output is of good quality. The applied methodology, data statistics, and accessibility are well documented. Such resources will be handy for future studies related to *P. azurea*. 

1. However, in the intro, besides description of life traits, it is not clear what kinds of research have been conducted and how this new genome could facilitate studies of *P. azurea* in the future.
2. The XY assemblies (18.98 Mb and 2.32 Mb repectively) are significantly smaller than those of autosomes (ranging from 151.39 Mb to 212.81 Mb). Knowing the difficulties when it comes to sex chromosome assembly, the authors should address how well their assembly represents the sequences of sex chromosomes. This is important especially considering it will serve as a reference for genomic studies to come.

Are sufficient details of methods and materials provided to allow replication by others?

Yes

Is the rationale for creating the dataset(s) clearly described?

Partly

Are the datasets clearly presented in a useable and accessible format?

Yes

Are the protocols appropriate and is the work technically sound?

Yes

Reviewer Expertise:

Evolutionary genetics, sex chromosome evolution, insect sex determination

I confirm that I have read this submission and believe that I have an appropriate level of expertise to confirm that it is of an acceptable scientific standard.
